# Pulmonary Mycobacterium abscessus Infection: A Pathogen in Disguise

**DOI:** 10.7759/cureus.46897

**Published:** 2023-10-12

**Authors:** Kiersten M Waugh, Rana Wajahat

**Affiliations:** 1 Infectious Diseases, Lincoln Memorial University DeBusk College of Osteopathic Medicine, Harrogate, USA; 2 Infectious Diseases, University Hospitals Portage Medical Center, Ravenna, USA; 3 Infectious Diseases, Western Reserve Hospital, Cuyahoga Falls, USA

**Keywords:** acid fast bacilli (afb), ‘pulmonary infection’, mycobacterium abscessus complex, nontuberculous mycobacterial infection, mycobacterium abscessus

## Abstract

The incidence of infections caused by nontuberculous mycobacteria (NTM) is rising around the world, and they are becoming increasingly difficult to treat due to the bacteria being drug-resistant to several antibiotics commonly used in practice. *Mycobacterium abscessus* complex (MABC) is particularly difficult to treat as it is one of the most antibiotic-resistant species and, therefore, has limited treatment options that are effective at clearing the infection. We present the case of an 81-year-old female who was diagnosed with *Mycobacterium abscessus* after several hospital admissions in a short period of time for similar complaints, and we will discuss her diagnosis and possible treatment plan. This case explores the challenges that physicians face in diagnosing NTM infections due to them mimicking several other conditions and raises the importance of having a high clinical suspicion.

## Introduction

Nontuberculous mycobacteria (NTM) are free-living organisms that can infect both immunocompromised and immunocompetent hosts, causing pulmonary or extrapulmonary disease. *Mycobacterium abscessus* complex (MABC) is one of over 190 species of NTM, categorized as a rapidly growing species and known for being among the most drug-resistant, making a timely diagnosis even more important and effective treatment challenging [1-3]. Out of the rapidly growing NTM, MABC is most associated with causing pulmonary infections and is particularly characteristic for infecting patients with underlying lung disease, including cystic fibrosis and bronchiectasis; however, there has also been an increasing number of infections in patients without any underlying lung conditions [4-6]. Diagnosis of NTM pulmonary infections is made by meeting the appropriate clinical presentation with pulmonary symptoms, having evidence on a chest radiograph or chest CT of nodular or cavitary opacities, and meeting microbiological criteria, which include either (1) positive cultures on two separate sputum samples, (2) having one positive from a bronchial lavage, or (3) mycobacterial histopathologic features on transbronchial or other lung biopsies such as stainable acid-fast bacilli or granulomatous inflammation along with one positive sputum or bronchial wash culture for NTM [7]. This case report discusses the clinical course of an 81-year-old female who was ultimately diagnosed with *Mycobacterium abscessus* after being treated for several other conditions along the way and discusses the appropriate management and treatment options.

## Case presentation

An 81-year-old female with a past medical history notable for chronic obstructive pulmonary disease (COPD) with no baseline oxygen requirement, congestive heart failure with preserved ejection fraction, coronary artery disease status post a prior coronary artery bypass graft as well as numerous percutaneous coronary interventions, peripheral vascular disease status post angioplasty and stenting to the right common iliac artery, and chronic kidney disease stage four, presented with shortness of breath. She was admitted several times during the course of a couple of months for similar complaints and was diagnosed and treated throughout this time for pneumonia, bronchiectasis, COPD exacerbations, and congestive heart failure exacerbations. Notably, the patient has had an extensive smoking history of one pack per day for 35 years; however, she quit in her early 50s. She is a native of New Orleans and is not at an increased risk for occupational lung disease.

**Figure 1:**
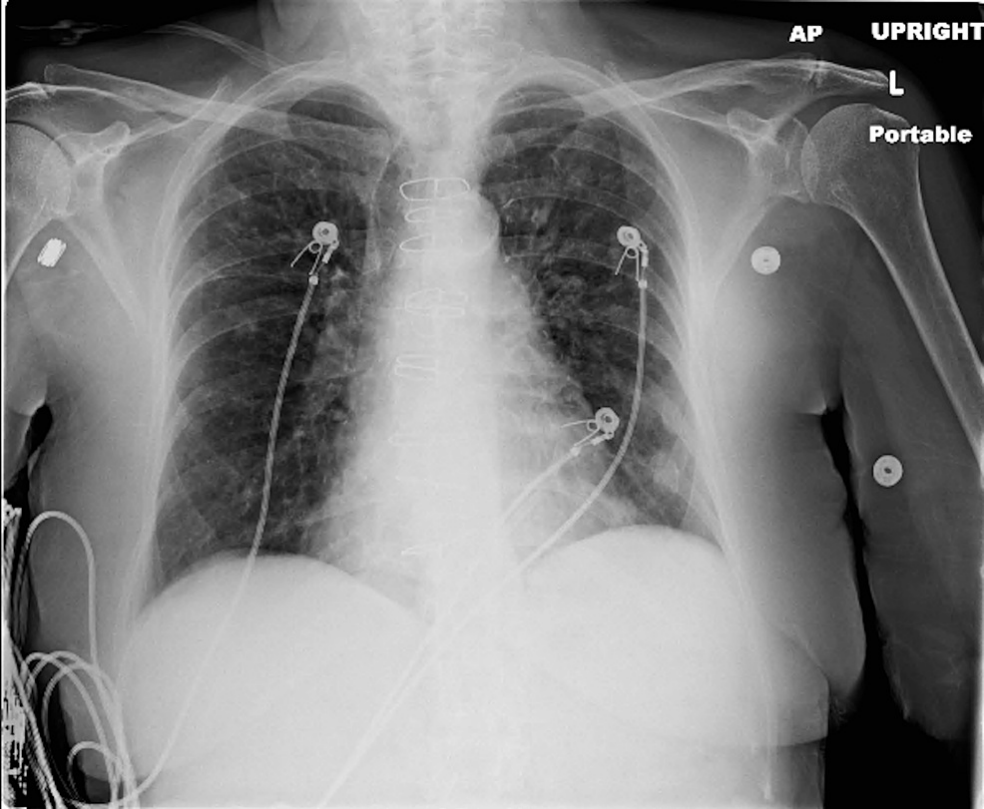
Initial chest X-rays were significant for patchy ground-glass opacifications.

During her first admission, she presented with shortness of breath with exertion and at rest that started worsening over the past day, accompanied by wheezing, orthopnea, and paroxysmal nocturnal dyspnea. Notably, the patient was seen outpatient one week prior for a productive cough of brown sputum that was treated with azithromycin, prednisone, and an albuterol inhaler for suspected acute bronchitis. On admission to the emergency department, she was hypertensive with a blood pressure of 183/93 mmHg, her heart rate was 66 bpm, her respiratory rate was 20 breaths/min, the temperature was 36.9 degrees Celsius, and she was saturating 94% on room air. Her white blood cell count was 8.0 x 10^9/L. Her initial chest radiograph revealed patchy ground-glass opacification suggesting pulmonary edema (Figure 1) along with a B-type natriuretic peptide value of 421 pg/mL, prompting her to be treated for pneumonia, congestive heart failure exacerbation and COPD exacerbation. She was placed on empirical antibiotic coverage, including Azithromycin and Ceftriaxone, given intravenous Lasix, and treated with Albuterol-Ipratropium nebulizers. Later on, during that hospital stay, she developed chest pain and subsequently received a percutaneous coronary intervention of her ostial diagonal artery with a drug-eluting stent. The patient was discharged home nine days after her admission, as she was in good condition. Upon discharge, the patient followed up with cardiology, who placed her on oral Lasix for volume overload, and pulmonology, who prescribed her Levaquin for community-acquired pneumonia; however, she did not start it as she was admitted again the following day.

**Figure 2:**
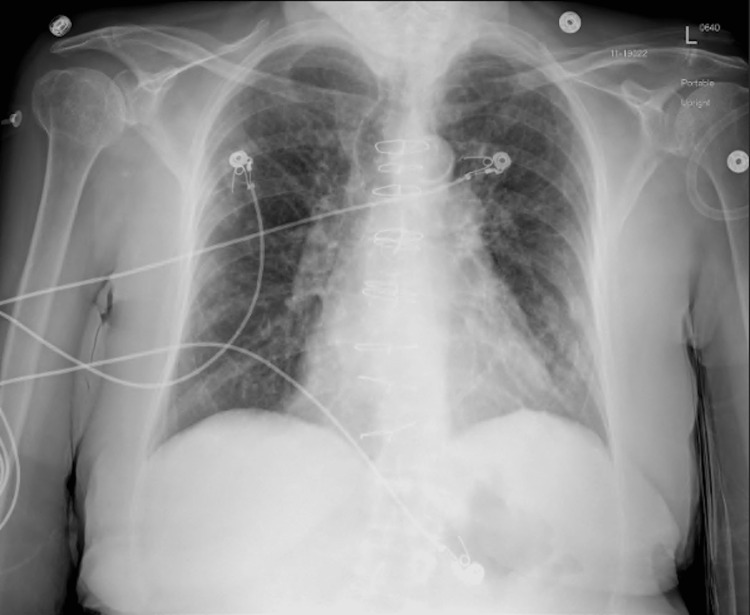
Chest X-ray on second admission was significant for worsening of interstitial infiltrates with upper lobe prominence.

**Figure 3:**
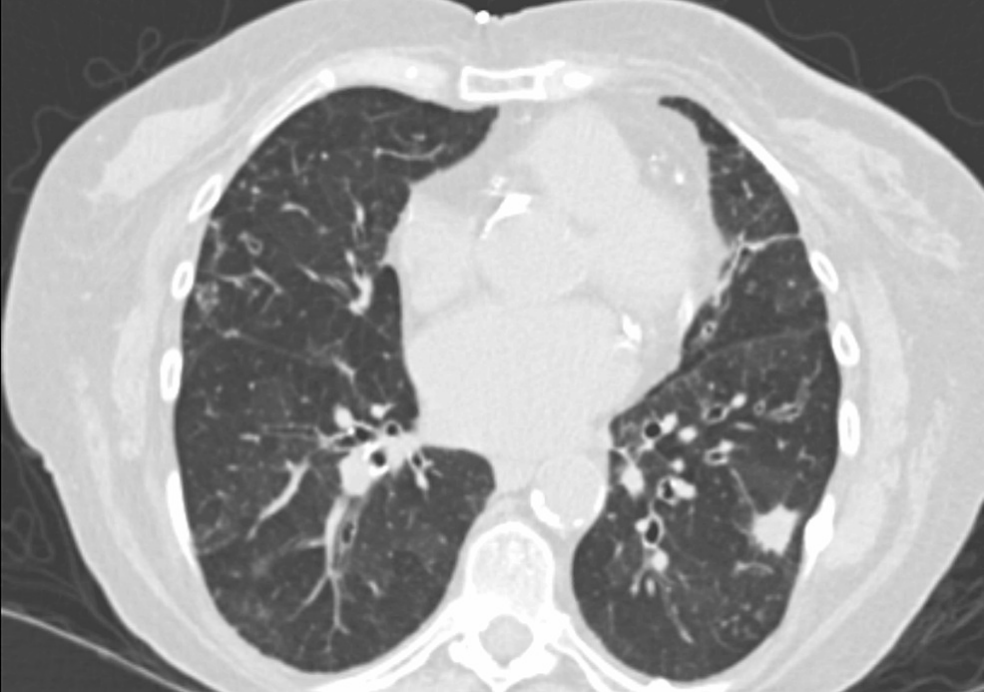
A non-contrast CT scan of the chest demonstrated patchy ground-glass opacities, bronchiectasis of moderate severity, and a left lower lobe nodule.

About two weeks after her first admission, the patient was admitted to the hospital again for worsening shortness of breath and cough. She was hypoxic on admission and required supplemental oxygen. Her white blood cell count was 9.6 x 10^9/L on admission, and the patient was afebrile. A chest radiograph revealed increased diffuse interstitial infiltrates compared to her previous studies, more prominent in the upper lobes with some mucus plugging (Figure 2). A noncontrast computed tomography (CT) scan of the chest revealed patchy reticulonodular infiltrates as well as ground glass opacities throughout the lungs with bronchiectasis, bronchial thickening, and mucoid impaction, suggesting an infectious cause. There were also spiculated nodules visualized in the left lower lobe measuring up to 1.6 cm, similar in size to the previous CT scan performed one month prior (Figure 3). She was started on empiric antibiotic therapy, including Vancomycin and Zosyn, for hospital-acquired pneumonia. The infectious disease team was consulted, and they added Doxycycline to her medication regimen. The following laboratory tests were ordered, including blood cultures, sputum samples for acid-fast bacillus smears and culture, respiratory viral panel, *Streptococcus pneumoniae* and *Legionella antigens*, *histoplasmosis* and *blastomycosis serologies*, *galactomannan*, and *Fungitell* assays. Blood cultures and procalcitonin came back within normal limits; therefore, Vancomycin and Zosyn were discontinued, and the patient was switched from Doxycycline to Azithromycin due to an allergy. Pulmonology was consulted to evaluate the need for bronchoscopy; however, it was ultimately decided that the patient would benefit most from medical management and pulmonary hygiene. The patient’s vitals remained stable, and it was recommended for her to be discharged with a prescription for an ANORO inhaler, Albuterol inhalers as needed, mometasone nasal spray, Mucinex, and mechanical vest therapy.

**Figure 4:**
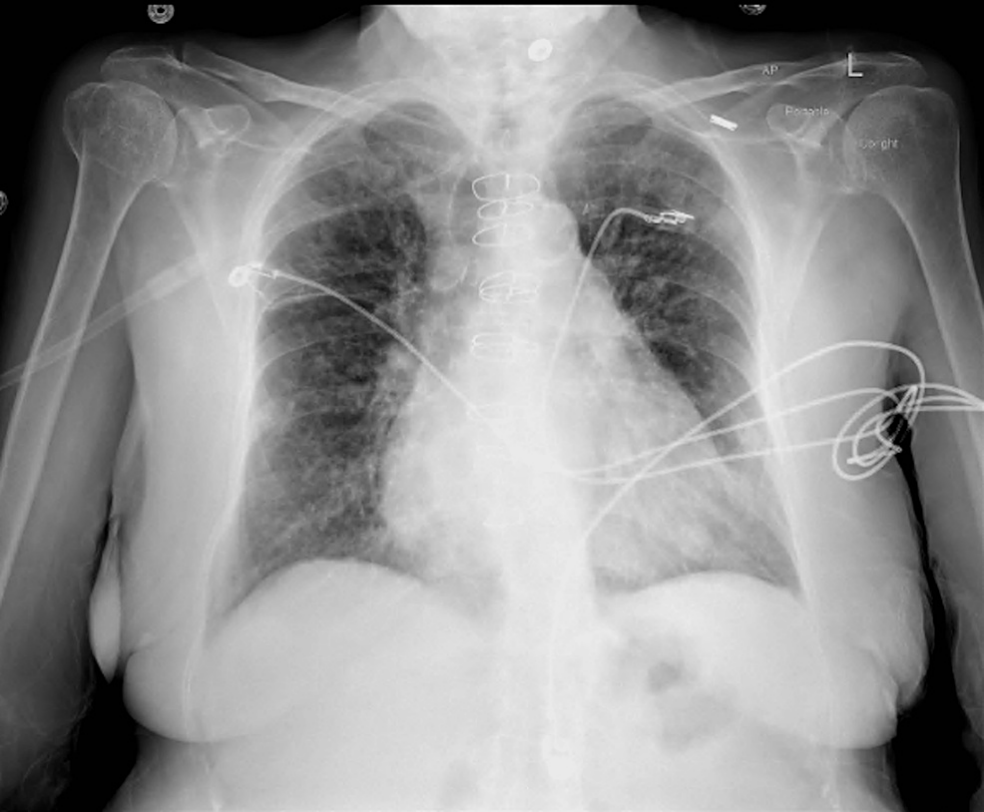
Chest X-ray on the third admission demonstrated the progression of reticulonodular infiltrates bilaterally.

The patient was admitted for the third time a little over a week later for shortness of breath and an increased cough, and on admission, her oxygen saturation was in the 70s. The patient denied any fevers, chills, chest pain, hemoptysis, nausea, or vomiting at this time. The chest X-ray was significant for worsening of the bilateral reticulonodular infiltrates seen previously on prior admissions (Figure 4). She was started on DuoNeb, solumedrol, and azithromycin. At this point, the acid-fast bacillus results from her previous admission had come back positive, but the species had not yet been identified; therefore, she was placed under airborne precautions. The infectious disease team ordered a quantiferon gold test for *Mycobacterium tuberculosis* and an MTB/RIF test, as well as repeat sputum samples for acid-fast bacillus testing. The quantiferon gold and MTB/RIF tests came back negative, making the suspicion for *Mycobacterium tuberculosis* infection low. Only one sputum sample was able to be obtained during this admission, and it came back positive again for acid-fast bacilli. The IDSA and ATS guidelines for nontuberculous mycobacterium species were now met due to there being two positive sputum cultures. The patient was discharged a few days later, when she was stable, and she was instructed to follow up with infectious disease as an outpatient.

Upon follow-up after hospitalization, treatment was held initially until the results of the speciation were finalized and ultimately identified as *Mycobacterium abscessus *on acid-fast bacilli culture. The patient was initially started on a medication regimen, including Minocycline, Linezolid, and Azithromycin; however, she subsequently developed thrombocytopenia, so her antibiotics were changed to Cefoxitin, Moxifloxacin, and Azithromycin. Later on, the moxifloxacin was discontinued due to its potential to increase uric acid and the patient’s reported history of gout, as well as increasing uric acid. The patient was then placed on a regimen, including IV Cefoxitin 2g every 48 hours, Azithromycin 500mg daily, and Minocycline 100 mg twice daily for two months. During the initial months of treatment, the patient was hospitalized again and developed a fungal urinary tract infection, likely due to her being on prolonged antibiotics. Along with the patient’s comorbidities and the challenges that those bring, she is also at an increased risk for further infections; therefore, she will be monitored closely during the treatment course. Repeat AFB sputum cultures were performed, and once negative results are obtained, treatment will be continued for an additional 12 months.

The patient has been followed as an outpatient and has been doing well clinically, with no further sputum production. Her repeat sputum cultures were negative, and she is now on an oral antibiotic regimen of Azithromycin 500mg daily and Cefdinir 300mg twice daily. She will continue to follow infectious disease as an outpatient every three months.

## Discussion

Along with *Mycobacterium avium complex* (MAC) and *Mycobacterium kansasii*, *Mycobacterium abscessus complex* (MABC) is one of the most frequent pathogens involved in NTM-associated pulmonary infections in the United States [4]. There are two commonly seen presentations of NTM lung infections: the upper lobe fibrocavitary form and the nodular bronchiectatic form [7]. The upper lobe fibrocavitary form is more common in older males with underlying lung disease, whereas the nodular bronchiectatic form is more common in nonsmoking postmenopausal women without lung disease [7]. This idea of NTM infections in elderly women without underlying lung disease was first described in the 1990s and was given the name “Lady Windermere Syndrome” [5]. One of the more recognized theories behind this phenomenon is that voluntary cough suppression leads to secretions collecting in the airways, forming a nidus for inflammation and resulting in chronic mycobacterial infection [2]. It is currently being studied as to whether there is a genetic predisposition for disease occurring in this group of patients. A study published in 2008 included 63 patients with pulmonary nontuberculous mycobacterial disease and found that, compared to control subjects, these patients were taller and leaner, with higher rates of scoliosis, pectus excavatum, mitral valve prolapse, and cystic fibrosis transmembrane conductance regulator (CFTR) mutations [1]. 

The most frequent presenting symptom of a MABC pulmonary infection is a chronic or recurring cough; however, the presentation is variable and can resemble many other common conditions [4]. Constitutional symptoms are common, including fatigue, dyspnea, fever, chest pain, and weight loss. Physical exam findings are also nonspecific but may include crackles, wheezes, and rhonchi [4]. In a study evaluating the radiographic signs of MABC pulmonary infection, it was found that the most common presentation on chest radiographs and computed tomography (CT) is bilateral reticulonodular opacities, followed by cavitary lesions and bronchiectasis [8]. When a patient has pulmonary symptoms and evidence of infection on radiographic studies, a MABC infection can be confirmed with microbiologic laboratory results. According to both ATS and IDSA guidelines, diagnosis can be confirmed with (1) positive cultures from at least two separate sputum samples, (2) positive cultures from at least one bronchial lavage, or (3) transbronchial or other lung biopsy with AFB or granulomatous inflammation and one positive culture from either a sputum sample or bronchial lavage [4,9]. In our case, the diagnosis of MABC infection was confirmed when two separate sputum cultures resulted in positivity for *M. abscessus*. Antibiotic susceptibility testing should be performed next, and for *M. abscessus* infections, it should include at least clarithromycin, cefoxitin, and amikacin [9]. It is recommended by the IDSA to wait until susceptibilities come back before beginning treatment instead of empiric antibiotic therapy to avoid creating further resistance [4].

Evaluation and diagnosis can be difficult due to patients having symptoms from concurring conditions, which makes it challenging to suspect a superimposed NTM infection. In our case, the patient’s symptoms resembled many common ailments, including pneumonia as well as chronic obstructive pulmonary disease and congestive heart failure exacerbations. Due to her ongoing symptoms despite treatment and recurrent admissions to the hospital, it was critical to have a high index of suspicion that another pathology was taking place, prompting the infectious disease team to take AFB sputum samples and discover the underlying infection. Even once the diagnosis has been made, it is recommended to treat any other ongoing lung conditions first before beginning treatment [8]. In addition, the goals of treatment should be taken into consideration, including the prognosis of the patient if treatment is initiated versus observation, especially since the cure rate is low [30%-50%] [7,9].

Antibiotic resistance among NTM species, especially MABC infections, which are resistant to typical antituberculosis drugs, makes treatment one of the greatest challenges. The first step when deciding on a treatment regimen for *M. abscessus* is determining whether the species is susceptible to macrolides, as macrolides are the mainstay of treatment. Treatment guidelines involve a long course of multiple drugs, divided into initial and continuation phases [9]. For patients with macrolide sensitivity, initial treatment should include a minimum of four weeks of azithromycin or clarithromycin plus one intravenous agent (amikacin, imipenem, cefoxitin, or tigecycline) and one oral agent (omadacycline, clofazimine, linezolid, or tedizolid) [10]. The continuation phase should include nebulized amikacin and an oral macrolide, along with one to three of the following: clofazimine, linezolid, minocycline or doxycycline, moxifloxacin or ciprofloxacin, and cotrimoxazole. For patients with demonstrated macrolide resistance, initial treatment includes a minimum of four weeks of intravenous amikacin, intravenous imipenem, and intravenous tigecycline. The continuation phase of treatment involves nebulized amikacin along with two to four of the following: clofazimine, linezolid, minocycline or doxycycline, moxifloxacin or ciprofloxacin, and cotrimoxazole [9]. In select patients, particularly those with severe disease or medication failure, surgical resection can be performed alongside medical therapy to aid in recovery [4]. The optimal time to switch from initial therapy to maintenance therapy is based on the toxicity and patient tolerance of long-term intravenous medication; however, typically, the switch occurs around three months. Treatment can be altered depending on patient tolerance and drug susceptibilities; however, it should be continued 12 months after the first negative culture in order for the infection to clear. Unfortunately, drug-related toxicity is very common [43.9%], especially liver injury, as well as infection recurrence. Therefore, treatment goals can be directed towards symptom improvement or regression of lung infiltrates rather than infection resolution [7].

## Conclusions

Our case emphasizes the pulmonary *Mycobacterium abscessus* complex as a particularly difficult infection to suspect and diagnose when the symptoms and presentation are vague and nonspecific and resemble many other more common conditions. This case highlights the importance of having a high clinical suspicion and including NTM infections in the differential, especially when a patient continues to be symptomatic despite appropriate treatment for underlying conditions and is repeatedly admitted to the hospital in a short period of time. Once appropriately diagnosed, due to high antibiotic resistance and low cure rates, it is important to discuss with the patient their goals of treatment before deciding whether to proceed with long-term therapy. The treatment regimens for this infection are long and complex, including multiple antibiotics continued over a long period of time. It is important to emphasize that many complications can occur during treatment, such as side effects and organ dysfunction caused by the drug regimens, as well as worsening of comorbidities and susceptibility to other infections. Overall, *Mycobacterium abscessus* is a challenging organism to detect and, ultimately, even more demanding and complicated to treat and eradicate.
